# Recipient myeloperoxidase-producing cells regulate antibody-mediated acute versus chronic kidney allograft rejection

**DOI:** 10.1172/jci.insight.148747

**Published:** 2021-07-08

**Authors:** Satoshi Miyairi, Daisuke Ueda, Takafumi Yagisawa, Daigo Okada, Karen S. Keslar, Kazunari Tanabe, Nina Dvorina, Anna Valujskikh, William M. Baldwin, Stanley L. Hazen, Robert L. Fairchild

**Affiliations:** 1 Department of Inflammation and Immunity, Lerner Research Institute, Cleveland Clinic, Cleveland, Ohio, USA.; 2 Department of Urology, Tokyo Women’s Medical University, Tokyo, Japan.; 3 Department of Cardiovascular & Metabolic Sciences, Lerner Research Institute, Cleveland Clinic, Cleveland, Ohio, USA.

**Keywords:** Transplantation, Macrophages, Monocytes, Neutrophils

## Abstract

Antibody-mediated rejection (ABMR) continues to be a major problem undermining the success of kidney transplantation. Acute ABMR of kidney grafts is characterized by neutrophil and monocyte margination in the tubular capillaries and by graft transcripts indicating NK cell activation, but the myeloid cell mechanisms required for acute ABMR have remained unclear. Dysregulated donor-specific antibody (DSA) responses with high antibody titers are induced in B6.CCR5^–/–^ mice transplanted with complete MHC-mismatched A/J kidneys and are required for rejection of the grafts. This study tested the role of recipient myeloid cell production of myeloperoxidase (MPO) in the cellular and molecular components of acute ABMR. Despite induction of equivalent DSA titers, B6.CCR5^–/–^ recipients rejected A/J kidneys between days 18 and 25, with acute ABMR, whereas B6.CCR5^–/–^MPO^–/–^ recipients rejected the grafts between days 46 and 54, with histopathological features of chronic graft injury. On day 15, myeloid cells infiltrating grafts from B6.CCR5^–/–^ and B6.CCR5^–/–^MPO^–/–^ recipients expressed marked phenotypic and functional transcript differences that correlated with the development of acute versus chronic allograft injury, respectively. Near the time of peak DSA titers, activation of NK cells to proliferate and express CD107a was decreased within allografts in B6.CCR5^–/–^MPO^–/–^ recipients. Despite high titers of DSA, depletion of neutrophils reproduced the inhibition of NK cell activation and decreased macrophage infiltration but increased monocytes producing MPO. Overall, recipient myeloid cells producing MPO regulate graft-infiltrating monocyte/macrophage function and NK cell activation that are required for DSA-mediated acute kidney allograft injury, and their absence switches DSA-mediated acute pathology and graft outcomes to chronic ABMR.

## Graphical abstract

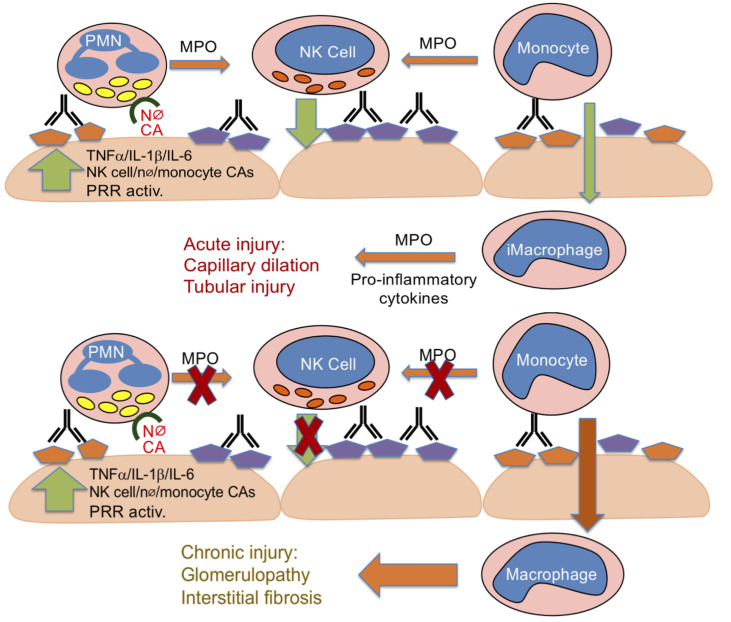

## Introduction

Antibody-mediated rejection (ABMR) continues to be an important cause of kidney graft injury and failure in patients transplanted to correct end-stage kidney disease (1–3). The incidence of acute ABMR can reach 12%, with substantial graft losses within the first 3 years after kidney transplant in patients having no detectable donor-specific antibody (DSA) prior to transplant (4, 5). Moreover, the incidence of acute ABMR can also promote the development of chronic kidney graft injury that is observed late after transplant and is the major cause of decreased late graft function and survival (1, 6–10). In contrast to the efficacy of therapies to treat T cell–mediated acute graft rejection, effective treatments to attenuate and reverse rejection mediated by DSAs are largely absent and continue to be a major focus of clinical trials in transplantation (11–13).

Acute ABMR of kidney grafts is typically characterized by microvascular inflammation, with neutrophil and monocyte margination in the peritubular capillaries with or without acute tubular injury (14). Clinical studies have reported that the intensity of monocyte/macrophage infiltration into kidney allografts during ABMR correlates with the magnitude of injury and graft outcomes (15–17). While these studies suggest that graft-infiltrating monocytes/macrophages may express proinflammatory functions that synergize with DSAs to promote antibody-mediated graft injury and rejection, specific functions of kidney graft–infiltrating myeloid cells that mediate this injury remain unclear. Myeloid cells are an important source of proinflammatory cytokines, such as TNF-α, IL-1β, and IL-6, but endothelial cells and other parenchymal cells in kidneys also produce these cytokines during inflammatory responses. One function that is characteristic of myeloid cells is the production of myeloperoxidase (MPO), a heme-containing enzyme that catalyzes the generation of reactive oxygen intermediates, including hypochlorous and hypobromous acids (18–20). MPO can make up to 5% of the total protein in neutrophils and is also produced by monocytes and some populations of tissue macrophages (21, 22). MPO plays a critical role in host defense to pathogens, including bacteria and yeast, in the pathogenesis of antineutrophil cytoplasmic antibody–associated (ANCA-associated) vasculitis, and it can also promote the development of chronic kidney and lung disease, cardiac infarction, and atherosclerosis (23–30). Despite this key role in tissue pathology, the role of MPO during donor-reactive immune responses to allografts, particularly the myeloid cell margination of tubular capillaries during ABMR of kidney grafts, has not been investigated.

One problem underlying the poor understanding of mechanisms leading to kidney graft injury during acute ABMR is the absence of preclinical models that reproduce the pathology and transcript signature observed in clinical kidney transplants experiencing ABMR. We have reported the dysregulated DSA responses evoked in response to heart and kidney allografts in CCR5^–/–^ mice with DSA titers that exceed those observed in wild-type recipients by >40-fold (31–33). Whereas most wild-type C57BL/6 recipients do not reject complete MHC-mismatched kidney allografts and maintain graft survival times of more than 100 days, B6.CCR5^–/–^ recipients typically reject these allografts within 17–30 days after transplant with histopathology virtually identical to that observed during acute ABMR of clinical kidney transplants, including the characteristic margination of neutrophils and macrophages in tubular capillaries, double contouring of glomerular capillaries, and diffuse C4d deposition (32). Furthermore, kidney allografts in CCR5^–/–^ recipients express the same transcripts, indicating NK cell activation during acute ABMR as those transcripts observed in clinical kidney grafts that have now become a diagnostic point for the pathology (34, 35). These molecular and histopathologic features of acute ABMR are dependent on B cells and DSAs, as they are absent in allografts from CCR5^–/–^ recipients depleted of B cells (32, 36). In the current study, we tested the role of MPO-producing myeloid cells on the cellular and molecular development of acute ABMR in kidney allografts. The results indicate a critical role for MPO in acute ABMR and the switch in DSA-mediated graft pathology to chronic ABMR in its absence.

## Results

### Recipient MPO is required for acute ABMR.

**Figure 1:**
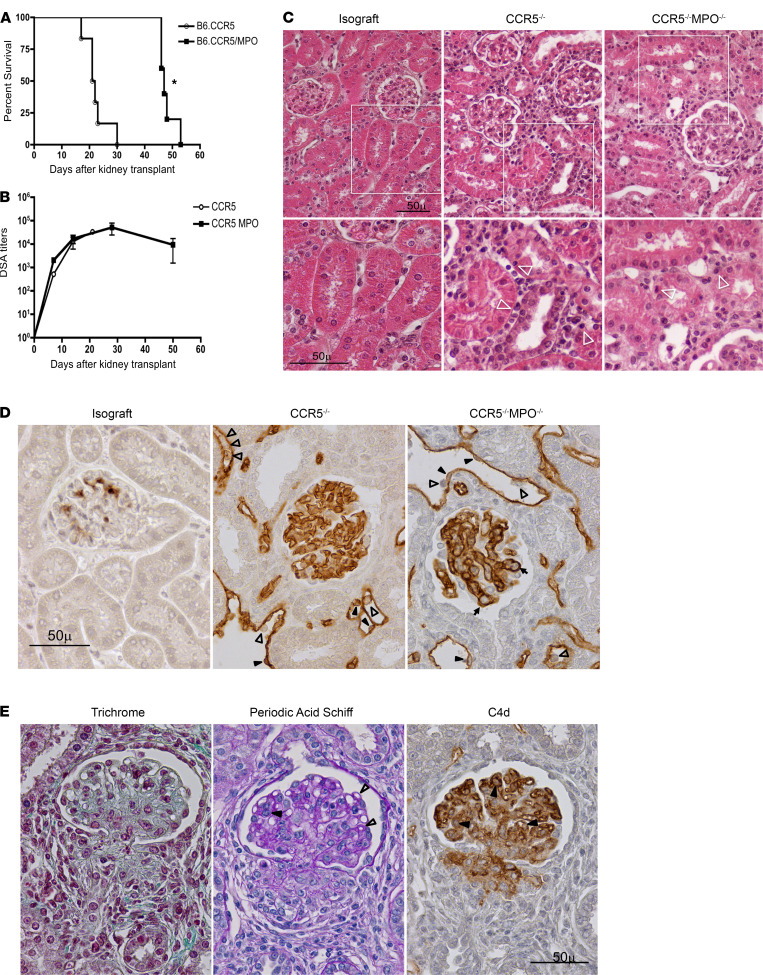
Switch from acute to chronic antibody-mediated kidney allograft rejection in the absence of recipient myeloperoxidase-producing cells. (**A**) Complete MHC-mismatched A/J (H-2^a^) kidney allografts were transplanted into groups of 6 B6.CCR5^–/–^ or 5 B6.CCR5^–/–^MPO^–/–^ (H-2^b^) mice. Native kidney nephrectomy was performed on day 4 after transplant. Survival of kidney grafts was followed by daily examination of animal health and confirmed by histopathologic evaluation of harvested grafts. Median survival time was day 22 in B6.CCR5^–/–^ recipients and day 47 in B6.CCR5^–/–^MPO^–/–^ recipients. *P < 0.002, Kaplan-Meier survival curves with log-rank statistics. (**B**) Sera from B6.CCR5^–/–^ and B6.CCR5^–/–^MPO^–/–^ recipients of A/J kidney grafts was obtained from individual recipients at the indicated times after transplant, and the titer of donor-reactive antibody (DSA) was determined. Data indicate mean titer for each graft recipient group ± SEM. (**C**) H&E staining of kidney isograft and allograft sections to assess of graft injury on day 14 after transplant. Consistent with histopathology of acute ABMR, margination of mononuclear cells into peritubular capillaries and infiltration into the tubules (white arrowheads) is observed in allografts from B6.CCR5^–/–^ recipients, and there is a marked decrease of this infiltration in allografts from B6.CCR5^–/–^MPO^–/–^ recipients. Allografts from both groups of recipients have DSA-mediated dilation of peritubular capillaries. Sections of C57BL/6 kidney isografts at day 14 are shown for comparison. (**D**) C4d staining of kidney isograft and allograft sections to assess graft injury on day 14 after transplant. Strong linear staining for C4d is evident in glomerular and peritubular capillaries of the allografts. Marginated mononuclear cells (white arrowheads) are present in dilated capillaries that are lined by endothelial cells with prominent nuclei (black arrowheads). Capillary loops of glomeruli are occluded by leukocytes and thickened (arrows). (**E**) Gomori’s trichrome, Periodic Schiff’s stain, and C4d staining of typical allograft sections from B6.CCR5^–/–^MPO^–/–^ recipients on day 48 after transplant indicate typical pathological features of chronic injury, including thickened capillary loops with doubled contours, mononuclear cells occluding capillary lumens, and periglomerular and peritubular fibrosis. Scale bar: 50 μm.

Since MPO is a primary inflammatory mechanism expressed by activated myeloid cells during tissue injury, the survival of complete MHC-mismatched A/J kidney grafts in B6.CCR5^–/–^ and B6.CCR5^–/–^MPO^–/–^ recipients was compared. As previously observed in B6.CCR5^–/–^ recipients, kidney allografts began to reject on day 18 after transplant, with all grafts rejected by day 30 and a median survival of 22 days (Figure 1A). In contrast, B6.CCR5^–/–^MPO^–/–^ recipients did not begin to reject allografts until day 47, with all rejected by day 52 after transplant and a median survival of 47 days (*P* < 0.002). Kinetics and titers of DSA induced in response to the allografts were identical in B6.CCR5^–/–^ and B6.CCR5^–/–^MPO^–/–^ recipients (Figure 1B), indicating that the prolonged allograft survival in B6.CCR5^–/–^MPO^–/–^ recipients was not due to attenuated DSA production. Examination of allografts harvested from B6.CCR5^–/–^ and B6.CCR5^–/–^MPO^–/–^ recipients on day 14 after transplant as DSA was nearing peak titers indicated diffuse C4d staining on glomerular and peritubular capillaries with the characteristic microvascular changes of dilated peritubular capillaries and marginated intracapillary monocytes in allografts from both sets of recipients (Figure 1, C and D), pathological findings that parallel those reported in clinical ABMR (2). In the absence of immunosuppression, Banff scores for interstitial inflammation at days 14–19 (*n* = 8/group) were 3 in grafts from both recipient groups (inflammation in >50% of unscarred cortical parenchyma), with macrophages and CD8^+^ T cells being the predominant cells; however, limited numbers of CD8^+^ T cells infiltrated tubules (t scores = 1.14 ± 0.79 and 1.13 ± 0.64 for the CCR5^–/–^ and CCR5^–/–^MPO^–/–^ recipients, respectively). Manifestations of ABMR were pronounced, with strong linear staining for C4d in glomerular and peritubular capillaries accompanied by capillary dilatation, endothelial cell swelling, and margination of mononuclear cells in all allografts at 14–19 days in both CCR5^–/–^ and CCR5^–/–^MPO^–/–^ recipients (Figure 1D). Glomerular capillary loops were occluded by leukocytes and thickened. However, the marked numbers of mononuclear cells marginated in peritubular capillaries and infiltrating tubules in allografts from B6.CCR5^–/–^ recipients were clearly decreased in the allograft interstitium and tubules from B6.CCR5^–/–^MPO^–/–^ recipients. At day 48 after transplant, A/J kidney allografts from B6.CCR5^–/–^MPO^–/–^ recipients exhibited typical pathological characteristics of chronic injury, including thickened capillary loops with double contours and mononuclear cells occluding capillary lumens as well as periglomerular and peritubular fibrosis (Figure 1E).

**Figure 2:**
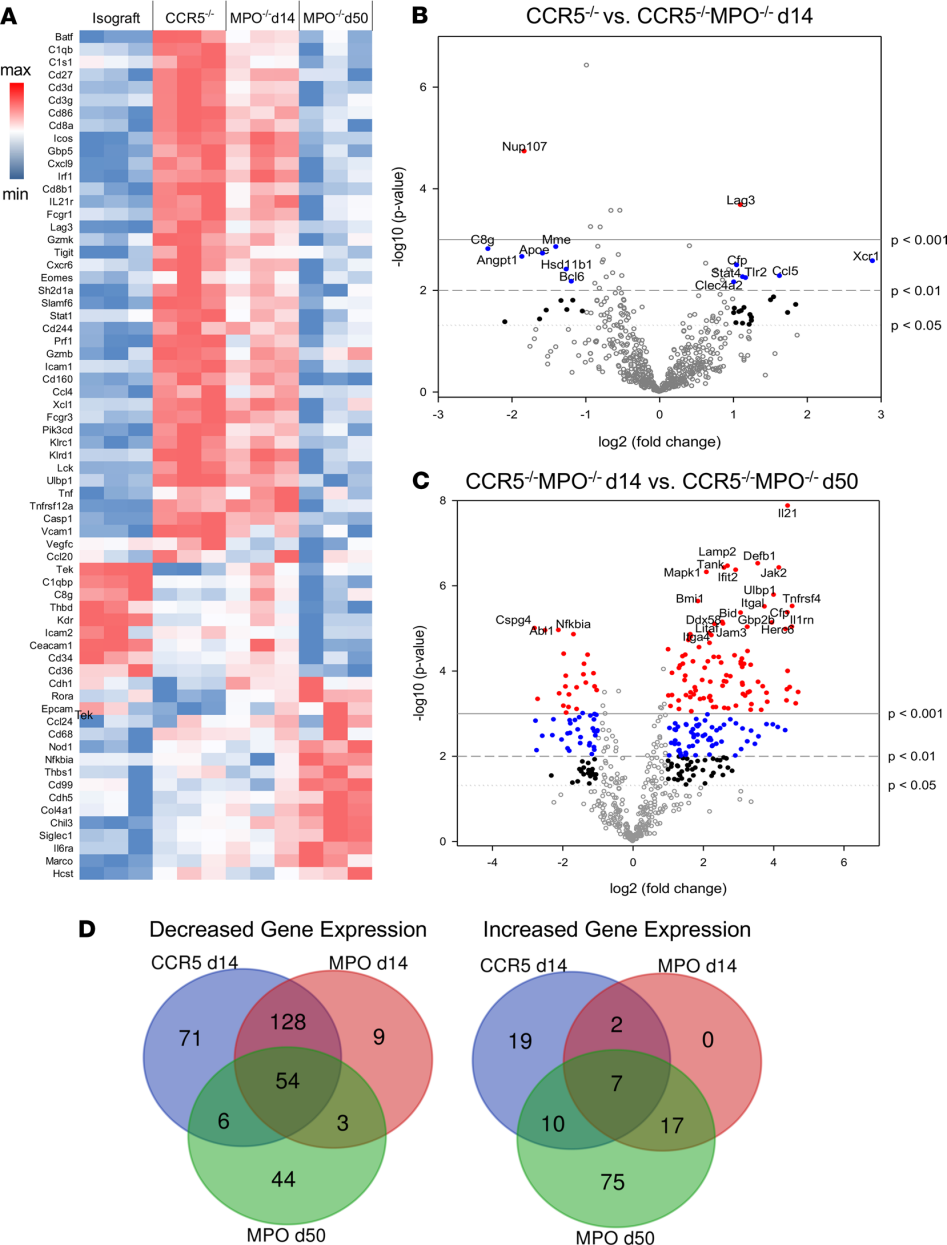
Different transcript landscapes in kidney allografts experiencing acute versus chronic ABMR. A/J kidney allografts were transplanted into B6.CCR5^–/–^ or B6.CCR5^–/–^MPO^–/–^ mice and harvested on day 14 or day 50 as indicated. As a control, C57BL/6 kidney isografts were harvested on day 14 from B6.CCR5^–/–^ recipients. (**A**) Graft RNA was isolated and analyzed by the NanoString nCounter platform using the Mouse PanCancer Immune Profiling panel, and a heatmap was generated from the top differentially expressed genes. (**B** and **C**) Volcano plots indicate increased and decreased genes expressed between (**B**) allografts from B6.CCR5^–/–^ recipients versus B6.CCR5^–/–^MPO^–/–^ recipients on day 14 after transplant and (**C**) allografts from B6.CCR5^–/–^MPO^–/–^ recipients on day 14 versus those from day 50 after transplant. In both volcano plots, open gray circles represent transcripts that are not changed between the 2 groups; solid black circles indicate differentially expressed genes (DEGs) with *P* < 0.05, 2-tailed *t* test; solid red circles indicate DEGs with *P* < 0.001, 2-tailed *t* test. The most highly significant DEGs are labeled on the plots. (**D**) Venn diagrams depicting numbers of shared and unique genes expressed in the allografts from B6.CCR5^–/–^ recipients on day 14 after transplant or from B6.CCR5^–/–^MPO^–/–^ recipients on day 14 or 50 after transplant compared with genes expressed in isografts on day 14 after transplant.

In order to test molecular differences correlating with these histopathologic differences, RNA isolated from B6 isografts and A/J kidney allografts from B6.CCR5^–/–^ and B6.CCR5^–/–^MPO^–/–^ recipients on day 14 after transplant and from long-term surviving allografts from B6.CCR5^–/–^MPO^–/–^ recipients on day 50 was interrogated by NanoString nCounter analysis (Figure 2, A–C). On day 14, expression of many inflammatory genes was increased in kidney allografts experiencing acute ABMR in B6.CCR5^–/–^ recipients versus kidney isografts, including those involved in leukocyte adhesion and activation of macrophages, T cells, B cells, and NK cells, and the expression of many of these genes was decreased in allografts harvested from B6.CCR5^–/–^MPO^–/–^ recipients on day 15 (Figure 2, B and D). In contrast, the expression of 9 genes, including the epithelial cell adhesion molecule, Epcam; the macrophage scavenger receptor, CD36; and genes involved in vascular endothelial recovery from injury was decreased in allografts from B6.CCR5^–/–^ recipients but increased in isografts and allografts from B6.CCR5^–/–^MPO^–/–^ recipients. By day 50, allografts in B6.CCR5^–/–^MPO^–/–^ recipients had decreased expression of CD36, the macrophage marker CD68, the TWEAK receptor (Tnfsrf12A), and Ulbp1, a stress induced ligand for NKG2D, but increased expression of genes encoding collagen, regulators of TLR and NF-κB signaling, intercellular adhesion molecules, and other molecules regulating cell adhesion to extracellular matrix proteins, including CD99 and thrombospondin (Figure 2, C and D). Collectively, these results indicate that despite equivalent titers of DSA, acute ABMR of kidney allografts is abrogated in the absence of recipient MPO-producing cells and instead progresses to unique gene expression changes over time after transplant that are associated with the development of chronic graft injury.

### Decreased activation of myeloid cells infiltrating kidney allografts in B6.CCR5^–/–^MPO^–/–^ recipients.

**Figure 3:**
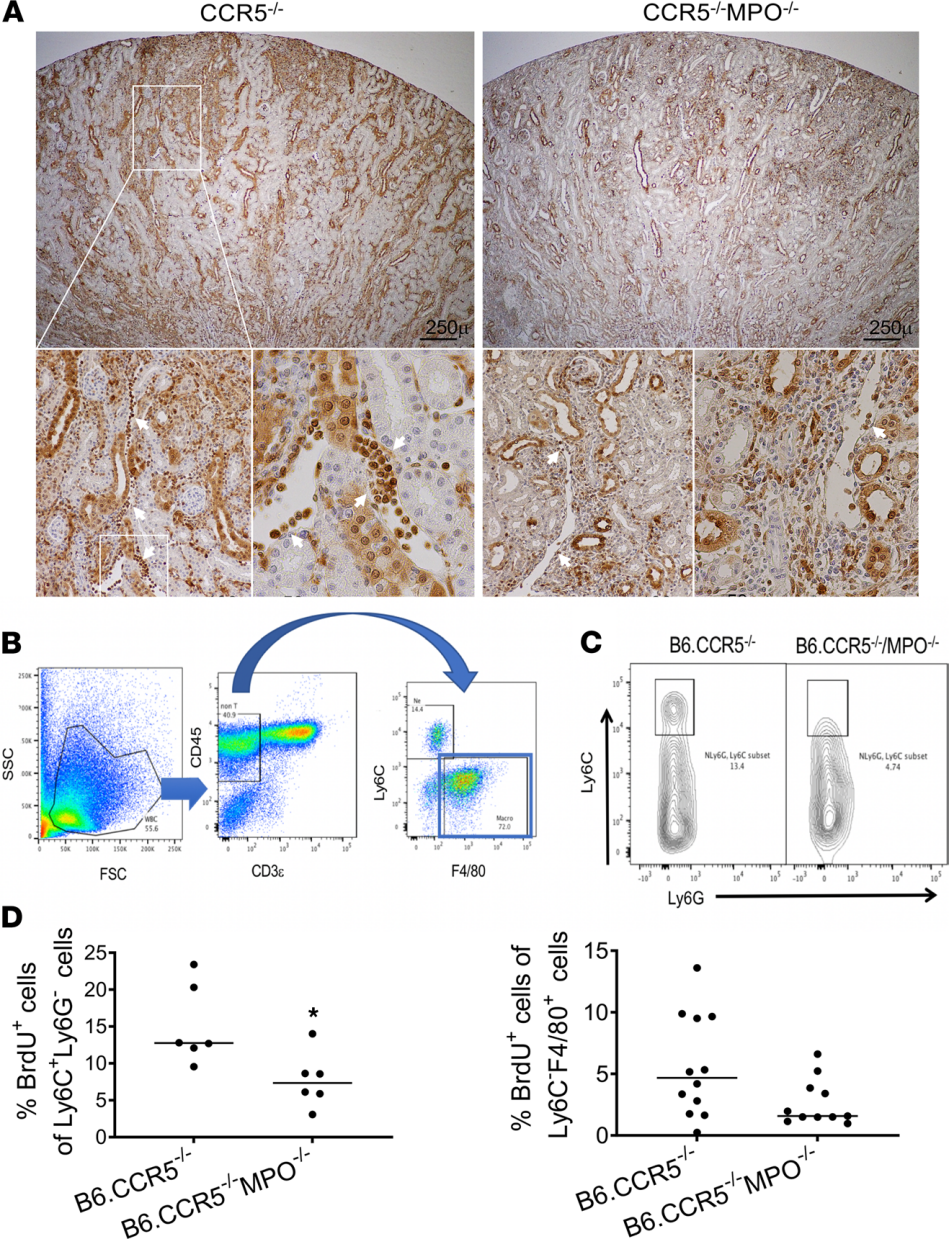
Marked differences in myeloid cells infiltrating A/J kidney allografts from B6.CCR5^–/–^ recipients versus B6.CCR5^–/–^MPO^–/–^ recipients. A/J kidney allografts were transplanted into groups of 5 B6.CCR5^–/–^ or B6.CCR5^–/–^MPO^–/–^ mice and harvested on day 15. (**A**) Representative sections of grafts that were fixed with methanol and stained to detect Mac-2^+^ macrophages in allografts from B6.CCR5^–/–^ recipients and their decrease in allografts from B6.CCR5^–/–^MPO^–/–^ recipients. Scale bar: 250 μm. (**B**) On day 15 after transplant kidney allografts were harvested and digested to obtain single-cell suspensions, and aliquots were stained with fluorochrome-labeled monoclonal antibody for flow cytometry analysis of cells expressing CD11c, Ly6C, F4/80, and Ly6G using the gating strategy indicated. (**C**) Representative analysis of Ly6C^+^F4/80^–^ monocytes within the allografts from each recipient group showing the presence of Ly6C^hi^ inflammatory monocytes infiltrating allografts from B6.CCR5^–/–^ recipients and their absence in allografts from B6.CCR5^–/–^MPO^–/–^ recipients. (**D**) Recipients were pulsed with BrdU on day 14 after transplant, and on day 15 grafts were harvested, digested, and aliquots of prepared single-cell suspensions were stained with antibodies and graft-infiltrating myeloid cells were gated to analyze the proliferation of Ly6C^+^F4/80^–^ monocytes and Ly6C^–^F4/80^+^ macrophages within the allografts. *P < 0.02, 2-tailed *t* test.

Clinical ABMR of kidney allografts is typically accompanied by margination of monocytes in the peritubular capillaries and macrophage infiltration into the graft that is reproduced during acute ABMR of kidney allografts in CCR5^–/–^ recipients (14–17). As these myeloid cells are a source of MPO, we compared their infiltration into kidney allografts in B6.CCR5^–/–^ and B6.CCR5^–/–^MPO^–/–^ recipients at the time DSA nears peak titers. Histochemical analysis of sections prepared from kidney allografts harvested on day 14 after transplant indicated the presence of Mac2^+^ macrophages in the peritubular capillaries of grafts from B6.CCR5^–/–^ recipients and a clear decrease in these cells in grafts from B6.CCR5^–/–^MPO^–/–^ recipients (Figure 3A). Allografts harvested from B6.CCR5^–/–^ and B6.CCR5^–/–^MPO^–/–^ recipients on day 15 after transplant were digested, and aliquots of prepared single-cell suspensions were stained with markers to distinguish T cells (CD3ε), monocytes (Ly6C^+^F4/80^–^), and macrophages (Ly6C^lo^F4/80^+^). More than 50% of the CD45^+^/non–T cells infiltrating allografts in both recipients were macrophages, with smaller populations of monocytes and neutrophils (Figure 3B). Macrophage and neutrophil numbers infiltrating the allografts from CCR5^–/–^MPO^–/–^ recipients were slightly, but not significantly, greater, and infiltrating monocyte numbers were slightly lower (data not shown). A Ly6C^hi^ inflammatory monocyte population observed in kidney allografts harvested from B6.CCR5^–/–^ recipients on day 15 after transplant was absent in allografts from the B6.CCR5^–/–^MPO^–/–^ recipients (Figure 3C). In addition, there were marked decreases in monocyte and macrophage proliferation within the allografts from B6.CCR5^–/–^MPO^–/–^ versus B6.CCR5^–/–^ recipients (Figure 3D).

**Figure 4:**
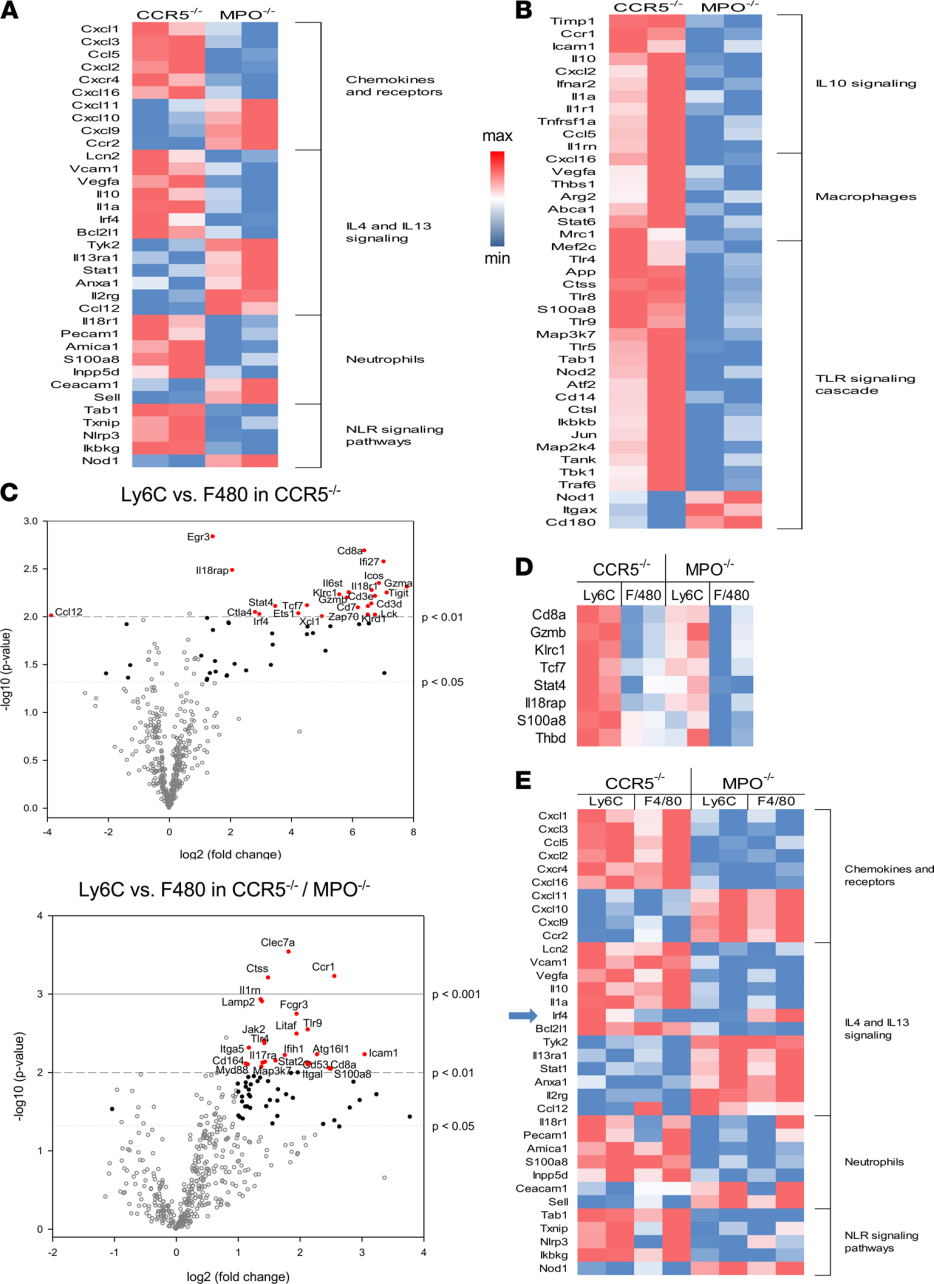
Differences in transcripts expressed by monocytes and macrophages infiltrating kidney allografts from B6.CCR5^–/–^ or B6.CCR5^–/–^MPO^–/–^ recipients. A/J allografts from groups of 2 B6.CCR5^–/–^ or B6.CCR5^–/–^MPO^–/–^ recipients were harvested on day 14 after transplant and were digested to obtain single-cell suspensions. Aliquots were stained with fluorochrome-labeled monoclonal antibody to identify the Ly6C^+^F4/80^–^ monocytes and Ly6C^–^F4/80^+^ macrophages that were purified by flow sorting. RNA from whole-cell lysates was analyzed by the NanoString nCounter platform using the Mouse Pan Cancer Immune Profiling panel, and heatmaps were generated from the top differentially expressed genes to compare transcript differences in the (**A**) Ly6C^+^F4/80^–^ monocytes and (**B**) Ly6C^–^F4/80^+^ macrophages infiltrating kidney allografts in B6.CCR5^–/–^ or B6.CCR5^–/–^MPO^–/–^ recipients. KEGG analyses of biological pathways were performed from differentially expressed genes in monocytes and macrophages isolated in the allografts from the 2 recipient groups. (**C**) Volcano plots indicate differentially expressed (up and down) genes (DEGs) expressed by purified Ly6C^+^F4/80^–^ monocytes versus Ly6C^–^F4/80^+^ macrophages infiltrating A/J allografts on day 14 after transplant from B6.CCR5^–/–^ (48 increased and 5 decreased DEGs) or B6.CCR5^–/–^MPO^–/–^ (64 increased and 1 decreased DEGs) recipients. In both volcano plots open gray circles represent transcripts that are not changed between the 2 groups; solid black circles indicate DEGs with *P* < 0.05, 2-tailed *t* test; solid blue circles indicate DEGs with *P* < 0.01, 2-tailed *t* test; solid red circles indicate DEGs with *P* < 0.001, 2-tailed *t* test; the most highly significant DEGs are labeled on the plots. (**D**) DEGs shared by the infiltrating monocytes versus macrophages in the 2 recipient groups were determined and are shown in the heatmap. (**E**) Heatmap comparing inflammatory genes expressed in monocytes versus macrophages infiltrating kidney allografts in B6.CCR5^–/–^ or B6.CCR5^–/–^MPO^–/–^ recipients. The expression of IRF4 by each population is noted by the blue arrow.

In order to directly test differences in monocytes and macrophages infiltrating A/J kidney allografts from B6.CCR5^–/–^ and B6.CCR5^–/–^MPO^–/–^ recipients, allografts were harvested on day 14 after transplant and were digested to prepare single-cell suspensions and the Ly6C^hi^/F4/80^–^ and Ly6C^lo^/F4/80^+^ populations were purified by flow sorting. RNA was isolated from each myeloid cell population and transcript landscapes were determined using NanoString analyses. The results indicated marked transcriptional differences in both Ly6C^hi^F480^–^ and Ly6C^lo^F4/80^+^ cells when comparing each population in kidney allografts from B6.CCR5^–/–^ and B6.CCR5^–/–^MPO^–/–^ recipients. For the graft-infiltrating Ly6C^hi^F480^–^ monocytes, there were distinct increases and decreases in expression of genes involved in the IL-4 and IL-13 as well as NLR signaling pathways and in chemokine and chemokine receptor gene expression (Figure 4A). For graft-infiltrating Ly6C^lo^F4/80^+^ macrophages, there were distinct differences in the increased expression of genes involved with cytokine signaling, macrophage differentiation, and TLR signaling in the graft-infiltrating macrophages from B6.CCR5^–/–^ and B6.CCR5^–/–^MPO^–/–^ recipients (Figure 4B). Since monocytes are stimulated to develop into macrophages during infiltration through the vascular endothelium, differences in gene expression were tested between Ly6C^hi^F480^–^ and Ly6C^lo^F4/80^+^ cells infiltrating kidney allografts in each recipient group (Figure 4C). For the transcript probes included in the test code set, allograft-infiltrating monocytes from B6.CCR5^–/–^ recipients had increased expression of 48 genes and decreased expression of 5 genes (>log_2_ fold change) compared with macrophages and infiltrating monocytes from B6.CCR5^–/–^MPO^–/–^ recipients, which had increased expression of 64 genes and decreased expression of 1 gene versus macrophages. When the differentially expressed genes (DEGs) between graft-infiltrating monocytes and macrophages in both recipient groups were compared, only 8 genes with increased expression were shared (Figure 4D). More frequently observed, however, was gene expression shared by both monocytes and macrophages isolated from the kidney allografts, and these were distinct to the allograft recipient (Figure 4E). One difference stood out, in that expression of IRF4 was increased in kidney allograft–infiltrating monocytes compared with macrophages from B6.CCR5^–/–^ recipients, whereas in allografts from B6.CCR5^–/–^MPO^–/–^ recipients infiltrating monocytes expressed low levels of IRF4 that were increased in the macrophages. Collectively, these results demonstrate the effect of recipient MPO-producing cells on the phenotype, transcriptional profiles, and functions of kidney allograft–infiltrating monocytes and macrophages during development of acute versus chronic ABMR.

### Decreased activation of allograft-infiltrating NK cells in B6.CCR5^–/–^MPO^–/–^ recipients.

**Figure 5:**
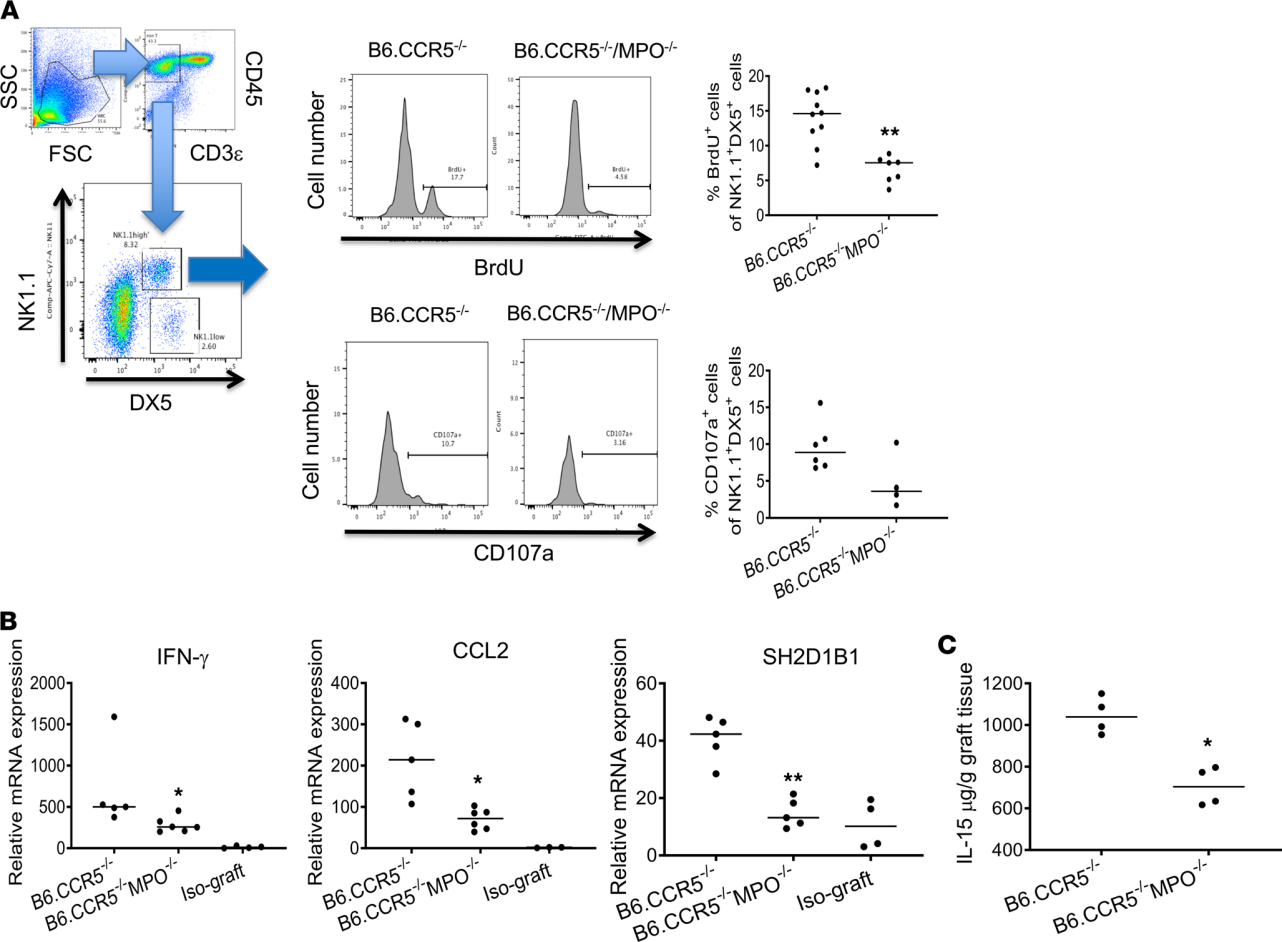
Decreased NK cell activation within A/J kidney allografts in the absence of recipient MPO-producing cells. A/J kidney allografts were transplanted into groups of 5 B6.CCR5^–/–^ or B6.CCR5^–/–^MPO^–/–^ mice. Recipients were pulsed with BrdU on day 14 after transplant. On day 15 grafts were harvested and digested to obtain single-cell suspensions, and aliquots were stained with fluorochrome-labeled monoclonal antibody for flow cytometry analysis. (**A**) Graft-infiltrating cells were gated as shown to analyze the CD3^–^NK1.1^+^DX5^+^ NK cells for expression of BrdU to indicate proliferating NK cells and for expression of CD107a to indicate cytolytic NK cells. **P < 0.001, 2-tailed *t* test. (**B**) Kidney allografts from groups of 5 B6.CCR5^–/–^ or B6.CCR5^–/–^MPO^–/–^ recipients were harvested on day 15 after transplant, and whole-cell RNA was isolated and analyzed by qPCR for expression of the indicated genes. Data indicate the mean ± SEM expression levels for each group. *P < 0.01, **P < 0005, 2-tailed *t* test. (**C**) Allografts from groups of 5 B6.CCR5^–/–^ and B6.CCR5^–/–^MPO^–/–^ recipients were harvested on day 15 after transplant and weighed, and protein homogenates of each graft were prepared. Quantity of IL-15 protein was determined by ELISA. Data indicate the mean ± SEM for each group per mg graft. *P < 0.05, 2-tailed *t* test.

**Figure 6:**
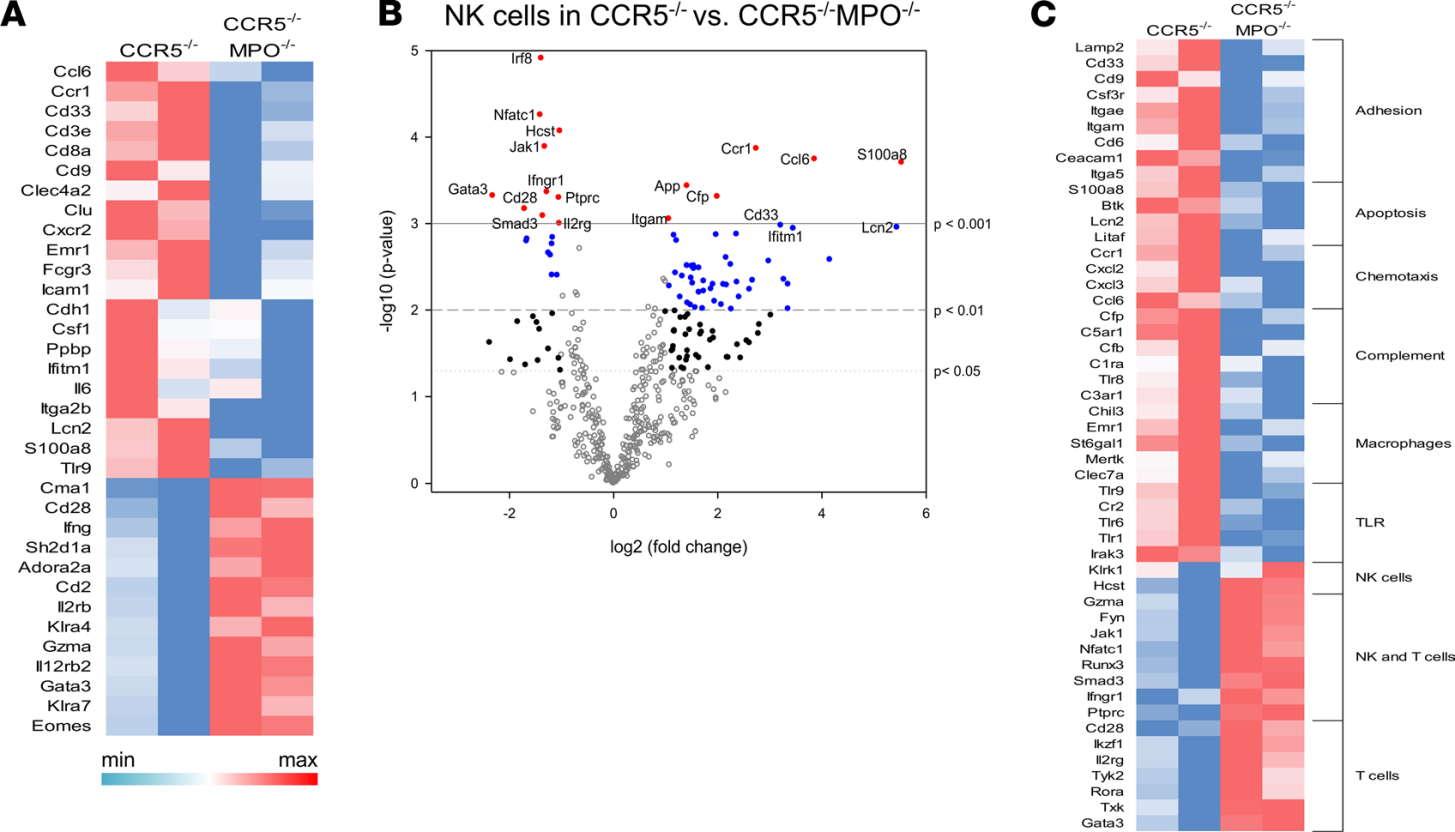
Differences in transcripts expressed by NK cells infiltrating kidney allografts from B6.CCR5^–/–^ or B6.CCR5^–/–^MPO^–/–^ recipients. A/J kidney allografts from groups of 2 B6.CCR5^–/–^ or B6.CCR5^–/–^MPO^–/–^ recipients were harvested on day 15 after transplant and were digested to obtain single-cell suspensions. Aliquots were stained with fluorochrome-labeled monoclonal antibody and analyzed by flow cytometry to identify the CD3^–^NK1.1^+^DX5^+^ NK cells that were then purified by flow sorting, and RNA from whole-cell lysates was analyzed by the NanoString nCounter platform using the Mouse PanCancer Immune Profiling panel. (**A**) A heatmap was generated from the top differentially expressed genes in NK cells isolated from allografts from B6.CCR5^–/–^ or B6.CCR5^–/–^MPO^–/–^ recipients. (**B**) Volcano plots indicate upregulated and downregulated genes expressed by purified NK cells in allografts from B6.CCR5^–/–^ or B6.CCR5^–/–^MPO^–/–^ recipients. Open gray circles represent transcripts that are not changed between the 2 groups; solid black circles indicate DEGs with *P* < 0.05, 2-tailed *t* test; solid blue circles indicate DEGs with *P* < 0.01, 2-tailed *t* test; solid red circles indicate DEGs with *P* < 0.001, 2-tailed *t* test. The most highly significant DEGs are labeled on the plots. (**C**) KEGG analyses of biological pathways from differentially expressed genes in the NK cells infiltrating allografts from the 2 recipient groups.

Our previous results indicated that acute ABMR of kidney allografts in CCR5^–/–^recipients requires NK cell infiltration and activation within the graft and is accompanied by infiltration of recipient myeloid cells and neutrophils (34, 35). To compare NK cell activation within kidney allografts in B6. CCR5^–/–^ and B6.CCR5^–/–^MPO^–/–^ recipients, allografts were harvested from groups of B6.CCR5^–/–^ and B6.CCR5^–/–^MPO^–/–^ recipients at the time DSA titers neared peak titers and were digested; the activation of graft-infiltrating NK cells was investigated by determining the levels of NK cell proliferation and expression of CD107a by flow cytometry. No difference was observed in NK cell numbers in the peripheral blood or within allografts from each of the recipient groups (data not shown). Pulsing of recipients with BrdU on the day before harvest indicated marked decreases in NK cell proliferation and expression of CD107a within allografts in B6.CCR5^–/–^MPO^–/–^ versus CCR5^–/–^ recipients (Figure 5A). These results are supported by the significant decreases in transcripts associated with NK cell activation, including IFN-γ, CCL2, and SH2D1B1, in allografts from B6.CCR5^–/–^MPO^–/–^ versus CCR5^–/–^ recipients (Figure 5B). Since IL-15 is required for maintenance and proliferation of NK cells (37, 38), IL-15 was quantitated in kidney allograft homogenates and was significantly decreased in allografts from B6.CCR5^–/–^MPO^–/–^ versus CCR5^–/–^ recipients (Figure 5C). NK cells were purified from graft digests by antibody staining and flow sorting, and isolated RNA was interrogated on the NanoString nCounter platform to compare the transcripts of NK cells infiltrating the kidney allografts in B6.CCR5^–/–^ and B6.CCR5^–/–^MPO^–/–^ recipients on day 15 after transplant (Figure 6). Consistent with the absence of NK cell activation, expression of genes encoding NK cell proinflammatory and cytotoxic function, integrin activation, and matrix degradation pathways were markedly decreased in graft-infiltrating NK cells in the absence of recipient MPO-producing cells.

### Recipient neutrophils are critical MPO-producing cells during acute ABMR of kidney allografts.

**Figure 7:**
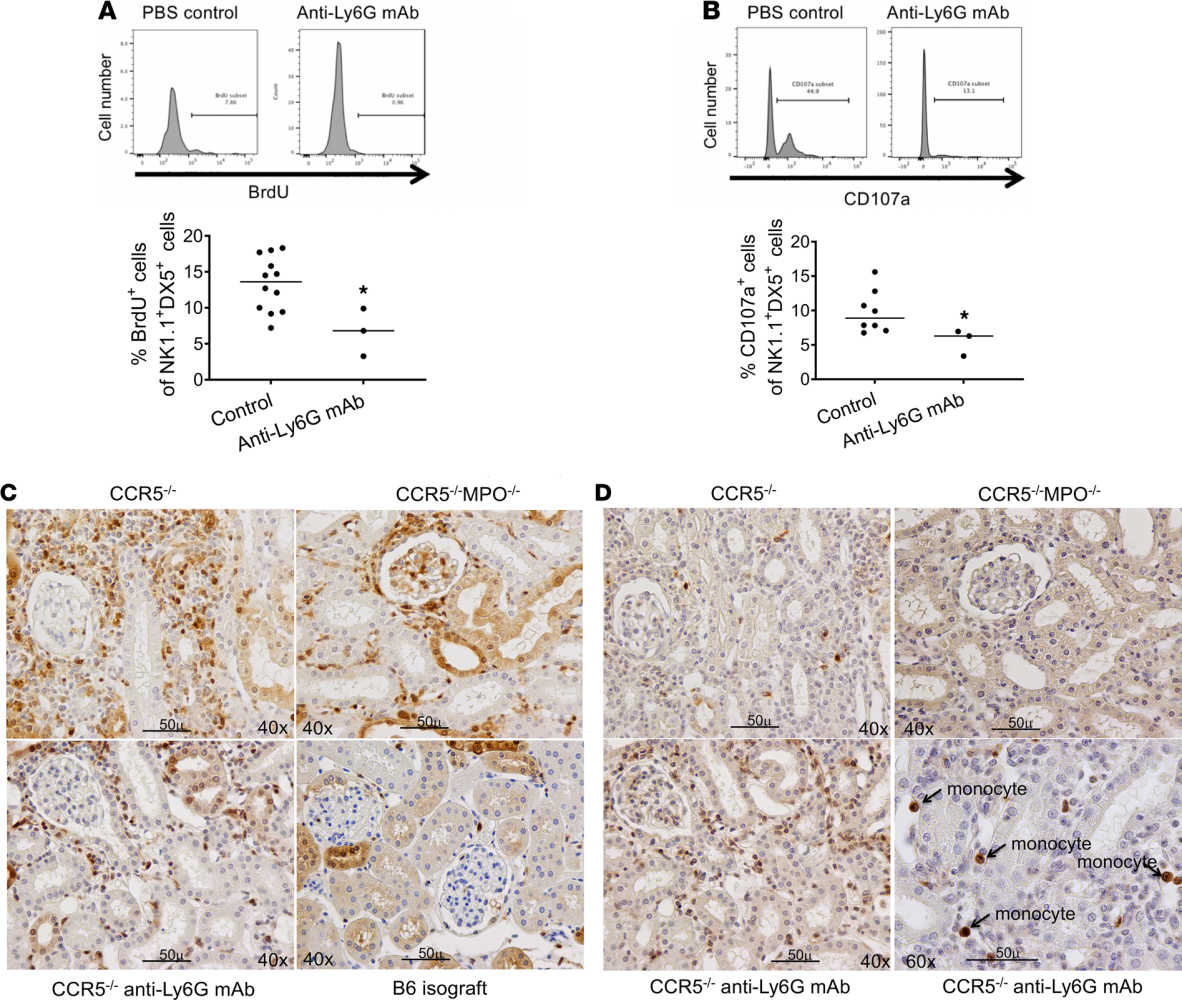
Neutrophil depletion abrogates acute antibody-mediated rejection of kidney allografts. A/J kidney allografts were transplanted into groups of 5 B6.CCR5^–/–^ mice that were treated with 250 μg anti-Ly6G monoclonal antibody or control rat IgG i.p. on days 10 and 12 after transplant. Recipients were pulsed with BrdU on day 14, and grafts were harvested on day 15 and digested to obtain single-cell suspensions. Aliquots were stained with fluorochrome-labeled monoclonal antibody for flow cytometry analysis. Graft-infiltrating cell populations were gated as in Figure 5 to analyze the CD3^–^NK1.1^+^DX5^+^ NK cells for expression of (**A**) BrdU and (**B**) CD107a. *P < 0.05, 2-tailed *t* test. (**C**) Representative sections of grafts fixed with methanol and stained with anti–Mac-2 antibody to detect Mac-2^+^ macrophages. Scale bar: 50 μm. Original magnification, ×40. (**D**) Representative sections of grafts fixed with methanol and stained with anti-MPO antibody to detect MPO-producing cells in the allografts. Scale bar: 50 μm. Original magnification, ×40.

Since neutrophils are a key source of MPO during infections and other tissue inflammation, we tested the effect of neutrophil depletion on the activation of NK cells within kidney allografts at the time of peak DSA titers in B6.CCR5^–/–^ recipients. Recipients were treated with neutrophil-depleting anti-Ly6G monoclonal antibody on days 10 and 12 after transplant, and NK cell activation within the allografts was tested 3 days later. Similar to the absent NK cell activation within kidney allografts from B6.CCR5^–/–^MPO^–/–^ recipients, neutrophil depletion abrogated NK cell proliferation within kidney allografts in B6.CCR5^–/–^ recipients as well as their expression of CD107a (Figure 7, A and B). Neutrophil depletion also resulted in decreased monocyte proliferation within the allografts and absent Ly6C^hi^ inflammatory monocytes (data not shown). Histologically, depletion of neutrophils at the time high DSA titers were achieved resulted in decreased Mac2^+^ macrophage infiltration into the interstitium of kidney allografts in CCR5^–/–^ recipients on day 14 after transplant (Figure 7C). Despite this decrease, neutrophil depletion increased the number of monocytes producing MPO within the capillaries of the kidney allograft in the CCR5^–/–^ recipients (Figure 7D).

## Discussion

Despite the clinical importance of acute ABMR of kidney allografts, mechanisms underlying this rejection remain poorly understood. We have utilized CCR5-deficient mice that produce high titers of DSA in response to kidney allografts and reject the grafts with virtually identical histopathology and expression of NK cell–associated transcripts as those observed during ABMR of clinical kidney transplants (32, 34, 36). The margination of monocytes, macrophages, and neutrophils into the tubular capillaries is a key diagnostic feature of acute ABMR, and the intensity of this infiltration is a portent of worse graft outcomes (14–17, 39, 40). In the current study, we report that the production of MPO by graft-infiltrating myeloid cells is required for NK cell activation and the progression of acute graft injury to ABMR. In the absence of MPO production, the myeloid cells switch from a proinflammatory function to functions promoting chronic allograft injury. While the DSA initiates graft inflammation by inducing the infiltration of a complex organization of NK cells and myeloid cells, it is the subsequent activation of the myeloid cells to express functions that generates the environment directing the progression of acute versus chronic graft injury.

During the development of ABMR, myeloid cell recruitment to the graft vasculature is initiated by DSAs binding to MHC ligands expressed by the graft endothelium to stimulate production of chemoattractants for neutrophils and monocytes, including IL-8, CXCL2, and CCL2 (41–46). This chemokine production has been shown in vivo by passively transferring anti-donor MHC antibody to allograft recipients and in vitro by stimulating human aortic endothelial cell cultures with class I HLA–specific antibody. The results of the current study suggest that, following DSA stimulation of the graft endothelium, and possibly other graft cells, innate immune components direct graft-infiltrating myeloid cell development to express functions that promote either acute or chronic allograft injury. At the time peak DSA titers are achieved there is a marked alteration in recruited monocyte function and their development to macrophages in the absence of recipient MPO-producing cells. These changes include phenotypic marker expression by the monocytes and decreases in graft-infiltrating monocyte and macrophage proliferation and in the transcripts expressed by each population. In the presence of MPO-producing cells and NK cell activation, monocytes contained a Ly6C^hi^ inflammatory monocyte population that was not observed in the absence of MPO-producing cells and NK cell activation. The function of the Ly6C^hi^ cells in the acute ABMR injury is unclear at this time and is a focus of ongoing studies. The Ly6C^hi^ inflammatory monocytes may be required for NK cell activation, and their absence may represent a default pathway to skew DSA-mediated graft injury from acute to chronic ABMR. This switch is possibly dependent on IRF4 expression, where graft-infiltrating monocytes express high levels and macrophages express low levels during acute ABMR in CCR5^–/–^ recipients and the monocytes express low levels of IRF4 and the macrophages express high levels during development of chronic ABMR in CCR5^–/–^MPO^–/–^ recipients. Several studies have reported the distinct roles of monocyte IRF4 expression during acute inflammatory processes and by macrophages during wound healing and chronic tissue injury (47–50).

Consistent with our previous studies (34, 35), a small proportion of NK cells within the A/J kidney allografts are activated to proliferate and express effector function, including CD107a, an indication of cytolytic activity. In the absence of recipient cells producing MPO, the presence of NK cells within the kidney allograft is detectable but their activation to proliferate and express CD107a is almost entirely absent. In contrast to genes expressing inflammatory effector functions, NK cells isolated in allografts from MPO-deficient recipients expressed transcripts involved with T cell activation. Similar to other instances where NK cells are not activated, kidney allograft survival is markedly extended, despite the presence of high DSA titers and the allografts develop chronic injury (35). This suggests that, in synergy with DSAs, MPO-producing myeloid cells promote NK cell activation to express functions mediating acute allograft injury and this activation program is not engaged in the absence of MPO-producing cells, regardless of the DSA in the absence of the MPO-producing cells, regardless of the DSA. Whether graft-infiltrating NK cells are inert in the absence of MPO-producing myeloid cells or express functions that promote the chronic graft injury is unclear and under investigation.

Neutrophils are typically the first inflammatory leukocytes recruited to inflammatory sites following release from the bone marrow and in solid-organ transplants are the first recipient-derived leukocytes to infiltrate grafts following revascularization (51–53). In contrast to their roles amplifying inflammation during IRI and T cell–mediated allograft rejection, the role of neutrophils, as well as MPO, in acute ABMR has not been well studied and is unclear. Neutrophil infiltration into kidney allografts in CCR5-deficient recipients is at high levels early after transplantation and then subsides with resolution of ischemia/reperfusion injury but increases again as DSA titers increase (34). This second wave of neutrophil infiltration appears to be independent of NK cell activation, as it is still observed when recipient NK cells are either depleted or inactivated (35). Since neutrophils are a key source of MPO (41), the studies were extended to test the effect of neutrophil depletion on the DSA-induced inflammatory components in kidney allografts during ABMR. Depletion of neutrophils as DSAs reach peak titers attenuates NK cell proliferation and expression of CD107a within the allografts. These decreases in NK cell activation are consistent with other studies, indicating neutrophil regulation of pathogen-mediated NK cell activation (54, 55). Neutrophil depletion in CCR5-deficient allograft recipients also obviated the generation of the Ly6C^hi^ monocytes and decreased monocyte proliferation within the kidney allografts but increased monocyte expression of MPO, implicating neutrophils as a regulatory mechanism of monocyte and macrophage function during ABMR. The abrogation of NK cell activation but increase in MPO-producing monocytes when neutrophils are depleted in allograft recipients producing high titers of DSA suggests that either neutrophil-derived MPO is directly required for NK cell activation to provoke acute ABMR or that additional neutrophil-mediated mechanisms are required to induce both NK cell and monocyte/macrophage activation during acute ABMR required for NK cell activation to provoke acute ABMR or that additional neutrophil-mediated mechanisms are required to induce NK cell and monocyte/macrophage activation during acute ABMR. The effect of neutrophil depletion on development of chronic injury and graft outcomes during these studies, however, was difficult to assess. Most CCR5^–/–^ kidney allograft recipients treated with the neutrophil-depleting antibody developed pyelonephritis by day 30 after transplant, although one of the allografts in neutrophil-depleted recipients survived more than 120 days (D. Ueda, data not shown).

The results of this study contribute new insights into the process of donor-specific antibody-mediated acute as well as chronic kidney allograft rejection. Defective function of graft-infiltrating myeloid cells to produce MPO abrogates NK cell activation, a key mechanism underlying acute ABMR. Most transplant patients maintain a course of immunosuppression to inhibit donor-reactive T cell responses that includes an antiproliferative drug and a calcineurin inhibitor. Whether these immunosuppressive drugs inhibit NK cell activation is not as clear, with in vitro and in vivo studies yielding unclear conclusions regarding their efficacy on NK cell activation (56–58). The results of the current study suggest that identification of myeloid cell targets will also attenuate acute ABMR. While this strategy is likely to improve graft function in the short term, it is likely to eventually lead to chronic kidney graft injury unless production of DSA can be eliminated. Overall, the results indicate that DSA binding to allograft MHC molecules organizes complex interactions of infiltrating recipient leukocytes, and their functions skew graft pathology to either acute or chronic injury. While these studies provide insights into these processes, they also identify the malleability of these innate immune players, and this may be useful in developing new diagnostics to follow the course of each of these graft pathologies and in developing new strategies to attenuate antibody-mediated acute and chronic kidney graft injury.

## Methods

### Mice.

A/J (H-2^a^), C57BL/6 (B6; H-2b), and B6.CCR5^–/–^ mice were obtained from The Jackson Laboratory and were maintained in the Lerner Research Institute Biological Resources Unit. B6.MPO^–/–^ mice, generated as previously reported, were crossed with B6.CCR5^–/–^ mice to generate B6.CCR5^–/–^MPO^–/–^ mice (59). Male mice (8–12 weeks) of age were used throughout this study.

### Kidney transplantation.

Murine orthotopic kidney transplant was performed using microsurgical methods reported by Zhang and colleagues (60). The left kidney was flushed with heparinized Ringer solution and harvested en bloc with the ureter and vascular supply from the graft donor. Then, the right native kidney of the recipient was removed and the donor artery and vein were anastomosed to the recipient abdominal aorta and inferior vena cava. Urinary reconstruction of the donor ureter to the recipient bladder was performed as previously reported (34). The remaining native left kidney was nephrectomized 4 days after transplantation so that recipient survival was completely dependent on the transplanted kidney function. Graft survival was assessed by daily observation of recipient health, and rejection was confirmed by histopathologic analysis. In some experiments, B6.CCR5^–/–^ kidney allograft recipients were treated i.p. with 250 μg control rat IgG or rat anti-mouse Ly6G monoclonal antibody A18 (BioXCell) on days 10 and 12 after transplant before sacrifice and analysis on day 15.

### Flow cytometry.

The harvested kidney graft was minced and digested by incubation with collagenase for 60 minutes at 37°C. A single-cell suspension was prepared and stained with antibodies and analyzed by flow cytometry. The following fluorochrome-conjugated antibodies were used for cell surface staining: rat anti-mouse CD3e (145-2C11) (eBioscience, Thermo Fisher Scientific), CD45 (BD Bioscience, Thermo Fisher Scientific), rat anti-mouse CD49b (DX5) (eBioscience, Thermo Fisher Scientific), rat anti-mouse NK1.1 (PK136) (BD Bioscience), rat anti-mouse Ly6G (BD Bioscience), rat anti-mouse Ly6C (BD Bioscience), rat anti-mouse F4/80 (eBioscience, Thermo Fisher Scientific), and rat anti-mouse CD107a (1D4B) (BioLegend). Analyses were performed on an LSR II flow cytometer (BD Bioscience), and data analyses were performed using FlowJo software (TreeStar Inc.). Proliferation of graft-infiltrating NK cells was assessed by BrdU labeling in vivo and then staining cells using BrdU Flow Kits (BD Bioscience). B6.CCR5^–/–^ allograft recipients were injected i.p. with 100 mL (1 mg) of BrdU solution the day before harvest. The following day, aliquots of single-cell suspensions from harvested grafts were prepared and stained with fluorescent anti-BrdU antibody and analyzed by flow cytometry. Isolation of graft-infiltrating cells by cell sorting was performed using FACS Aria after labeling with antibodies as described above, and the sorted cell populations were resuspended in PBS and washed before isolation of RNA.

### Measure of DSA titers.

Titers of donor-reactive IgG antibody in recipient serum were assessed by a flow cytometry–based analysis as previously reported (32–34). Briefly, the mean channel fluorescence of each dilution of each serum sample was determined, and the dilution that returned the mean channel fluorescence to the level observed when A/J thymocytes were stained with a 1:4 dilution of normal C57BL/6 mouse serum was divided by 2 and reported as the titer.

### RNA extraction, cDNA synthesis, and quantitative analysis of gene expression.

Total RNA was isolated from snap-frozen kidney graft tissue using RNeasy Mini Kits (QIAGEN), and 1 μg was reverse transcribed using High-Capacity cDNA Archive Kits (Applied Biosystems, Thermo Fisher Scientific). Ten microliters of each cDNA was used for quantitative PCR with TaqMan Fast Universal Master Mix and TaqMan primer sets. All PCR reactions were done in duplicate on a 7500 Fast Real-Time PCR System (Applied Biosystems, Thermo Fisher Scientific) and quantified using the ddCt method and primers for IFN-γ (Mm01168134_ml), CCL2 (Mm00441242_ml) and SH2D1B1 (Mm04210368_ml), with MRPL32 (00777741_SH) as the reference gene (Applied Biosystems, Thermo Fisher Scientific).

### Immunohistochemistry analysis of kidney graft tissue.

Kidney grafts were harvested and fixed in acid methanol (60% methanol and 10% acetic acid). Paraffin-embedded sections (5 μm) were subjected to high temperature antigen retrieval and paraffin removal in Trilogy (Cell Marque) in a pressure cooker. Endogenous peroxidase activity was eliminated by incubation with 0.03% H_2_O_2_ for 10 minutes, and nonspecific protein interactions were inhibited by incubation with serum-free protein block (DAKO). The slides were then stained using H&E and Gomori’s Trichrome (Richard-Allan Scientific) and with the following primary antibodies: rat monoclonal antibody against mouse Mac-2 (clone M3/38; Cedarlane Laboratories), rabbit monoclonal anti-MPO antibody (clone EPR20257; Abcam), and rabbit polyclonal antiserum to α-smooth muscle actin (Abcam). Primary antibodies were visualized using rat or rabbit on mouse HRP-Polymer Kits (Biocare Medical) followed by DAB and counterstained with hematoxylin. Slides were viewed by light microscopy, and images captured using ImagePro Plus (Media Cybernetics).

### Nanostring nCounter analyses.

One hundred ng of RNA from kidney graft tissue was used in each Nanostring hybridization. Freshly purified graft-infiltrating NK cells, monocytes, or macrophages were resuspended in a lysis solution of 1 part RLT buffer from the QIAGEN RNeasy Mini Kit to 2 parts nuclease-free water, and 4.5 μL of the lysate was added directly to the NanoString hybridization. Gene expression was measured using the mouse PanCancer Immune Profiling Panel. Log_2_ normalized counts and expression ratios were generated using nSolver version 4.0 and advanced analysis version 2.0.

### Statistics.

Data analysis was performed using GraphPad Prism 5.0 software. Comparisons of kidney allograft survival between groups were analyzed using Kaplan-Meier survival curves and log-rank (Mantel Cox) statistics. Statistical differences between 2 experimental groups were analyzed using 2-tailed *t* tests. *P* values of less than 0.05 were considered significant. Error bars represent SEM for each experimental group.

### Study approval.

All animal procedures were approved by the Cleveland Clinic Institutional Animal Care and Use Committee.

## Author contributions

SM and DU helped equally with experimental design, performance of kidney transplants and experiments analyzing graft-infiltrating cells, and with preparation of figures. SM began the project and was key in generating the data presented in Figures 1–6 and was therefore listed first in the authorship list; DU was key in generating the data presented in Figures 3 and 7. TY and DO performed kidney transplants. KSK performed NanoString nCounter analysis of RNA and presentation of the data. KT helped with experimental design and data interpretation. ND performed staining of graft biopsy sections. WMB performed analysis of stained graft biopsy sections. KT, AV, WMB, SLH, and RLF participated in experimental design, data interpretation, and preparation of the manuscript.

## Version 1 06/03/2021

In-Press Preview

## Version 2 07/08/2021

Electronic publication
